# A High Precision Method for Quantitative Measurements of Reactive Oxygen Species in Frozen Biopsies

**DOI:** 10.1371/journal.pone.0090964

**Published:** 2014-03-06

**Authors:** Kirsti Berg, Madelene Ericsson, Mikael Lindgren, Håkan Gustafsson

**Affiliations:** 1 Dept. of Cancer Research and Molecular Medicine, Norwegian University of Science and Technology, Trondheim, Norway; 2 Dept. of Medical Biosciences, Physiological Chemistry, Umeå University, Umeå, Sweden; 3 Dept. of Physics, Norwegian University of Science and Technology, Trondheim, Norway; 4 Dept. of Physics, Chemistry and Biology, Linköping University, Linköping, Sweden; 5 Dept. of Biomedical Engineering (MTÖ), County Council of Östergötland, Radiation Physics, Dept. of Medicine and Health Sciences, Linköping University, Linköping, Sweden; National Institutes of Health, United States of America

## Abstract

**Objective:**

An electron paramagnetic resonance (EPR) technique using the spin probe cyclic hydroxylamine 1-hydroxy-3-methoxycarbonyl-2,2,5,5-tetramethylpyrrolidine (CMH) was introduced as a versatile method for high precision quantification of reactive oxygen species, including the superoxide radical in frozen biological samples such as cell suspensions, blood or biopsies.

**Materials and Methods:**

Loss of measurement precision and accuracy due to variations in sample size and shape were minimized by assembling the sample in a well-defined volume. Measurement was carried out at low temperature (150 K) using a nitrogen flow Dewar. The signal intensity was measured from the EPR 1^st^ derivative amplitude, and related to a sample, 3-carboxy-proxyl (CP•) with known spin concentration.

**Results:**

The absolute spin concentration could be quantified with a precision and accuracy better than ±10 µM (k = 1). The spin concentration of samples stored at −80°C could be reproduced after 6 months of storage well within the same error estimate.

**Conclusion:**

The absolute spin concentration in wet biological samples such as biopsies, water solutions and cell cultures could be quantified with higher precision and accuracy than normally achievable using common techniques such as flat cells, tissue cells and various capillary tubes. In addition; biological samples could be collected and stored for future incubation with spin probe, and also further stored up to at least six months before EPR analysis, without loss of signal intensity. This opens for the possibility to store and transport incubated biological samples with known accuracy of the spin concentration over time.

## Introduction

Quantification of reactive oxygen species (ROS) in biological samples such as cell suspensions, blood or biopsies and in whole organisms is of large medical and biological interest. Detection and quantification of ROS can be performed by indirect methods, such as observations of chemical changes caused by ROS or by direct quantification of the amount of ROS [1]–[3]. There are other optical techniques for measuring ROS e.g., spectrophotometric measurement of cytochrome C reduction, fluorescence quantification of dihydroethidium- DHE and methods for assessment of tissue oxidative damage [3]–[5]. One of the best available methods for quantification of the amount of ROS in tissues is quantitative electron paramagnetic resonance (EPR) [2], [3].

A new generation of spin probe molecules (cyclic hydroxylamine’s) [6]–[9], and the high sensitivity of modern X-Band EPR spectrometers fitted with super high Q resonators, have made it possible to measure very low concentrations of radicals and other paramagnetic species both in vitro and in biopsies. By using the spin probe molecule CMH (1-hydroxy-3-methoxycarbonyl-2,2,5,5-tetramethylpyrrolidine) it is possible to detect several radicals and reactive oxygen species such as; the superoxide ion, peroxyl radical, peroxynitrite and nitrogen dioxide, however CMH does not react with nitric oxide or hydrogen peroxide. By using the CMH spin probe the main detected molecule is the superoxide ion and not ROS in general.

[4]–[6], [8]. ROS is an abbreviation of a large class of chemicals and care must therefore be taken when choosing spin probe for the molecule of interest. When it comes to quantitative EPR, and especially measurement of radicals in wet samples such as biopsies, water solutions and cell cultures, the use of common techniques (e.g. flat cells, tissue cells and various capillary tubes) may often result in loss of accuracy and precision. A large number of experimental parameters influence the signal intensity of an obtained EPR spectrum. Without a proper and detailed experimental protocol the differences in EPR signal intensity will depend on a variety of different factors such as microwave settings and cavity matching, sample position, sample size and shape, rather than a simple relation to total concentration of radicals in the sample. Even very small differences in sample position and shape may induce large changes in resonator quality factor (Q value), i.e. spectrometer sensitivity, between different measurements with subsequent loss of accuracy and precision. This can partly be compensated for by the use of an internal reference sample such as Mn^2+^/MgO or ruby [10], [11]; but signal normalisation to a reference sample or to the resonator Q value is associated with an additional uncertainty. Precise measurement of the loaded resonator Q value may replace normalisation to a reference sample and can be used for reference free quantitative EPR with an uncertainty of less than 10% (k = 1) [12], [13]. However, it is sometimes desired to observe even very small differences in generation of ROS between different biological samples. The development of a measurement protocol with higher precision and accuracy than what can be achieved using common techniques such as flat cells, tissue cells and various capillary tubes is therefore needed.

A method to improve accuracy and precision is central in the related field of EPR dosimetry where radiation dose is measured for medical applications in radiation therapy of cancer [14], [15]. The experiences from recent developments in EPR dosimetry can be used to further improve accuracy and precision in quantitative EPR of radicals in wet samples such as biopsies, water solutions and cell cultures. It has recently been showed that high precision and accuracy can be achieved in EPR dosimetry without the use of an external reference [16], [17], but instead by precisely control sample size and sample position, in addition to the use of a sample tube with a homogenous wall thickness. Here we want to develop this further to a high precision method, but yet versatile and manageable, for quantitative measurement of primarily the superoxide ion and other radicals that oxidase CMH in fresh or frozen biopsies, based on measurements using frozen “tablets” measured at 150 K with a special-designed Dewar.

The proposed method was tested for quantification of reactive oxygen species in a series of relevant biopsies (myocardium and aortic plaques from rodents). The time dependence of the EPR signal obtained after incubating biopsies with CMH was also studied with the aim to study the possibility to transport incubated samples on dry ice and the possibility of repeated measurements after several months of storage.

## Materials and Methods

All solutions were solved in freshly made (using bi-distilled water) and filtered (200 mesh) Krebs Hepes Buffer (KHB) (NOX-7.6. Noxygen, Germany). 10 mM stock solution of the CMH spin probe: 1-hydroxy-3-methoxycarbonyl-2,2,5,5-tetramethylpyrrolidine (CMH) (NOX-2, Noxygen, Germany) was freshly made and dissolved in KHB containing 25 µM deferoxamine methanesulfonate salt (DF) (NOX-9, Noxygen, Germany) and 5 µM sodium diethyldithiocarbamate trihydrate (DETC) (NOX-10, Noxygen, Germany) under constant bubbling of N2 (g), to keep an oxygen free atmosphere.

Rat (male, Wistar, Taconic, DK) myocardium (N = 16) or mouse (C57Bl/6 wild type (N = 6) and ApoE^−/−^ (N = 2)) aortic endothelium were rapidly frozen in liquid N_2_ immediately after collection, and thereafter stored at −80°C. Tissue was sampled as part of other experiments (data not shown). All animal experiments were approved by the Committee on the Ethics of Animal Experiments of the University of Trondheim (Permit Number: 12947-2006), and performed in accordance with the local Animal welfare act, which closely conforms to the NIH guidelines.

For incubation of biopsies in spin probe, frozen tissue samples as small as 6 µg were quickly chopped in 147 µL oxygen free KHB and added to a micro centrifuge tube with cap. 3 µL oxygen-free spin probe (10 mM CMH) was added to final concentration: 200 µM CMH. The tube was capped before immediately incubating in a shaking water bath (37°C). After exactly 60 min, the test tube was centrifuged (quick spin), and exactly 100 µL of the solution was transferred to a 1 mL de-capped syringe and snap frozen in N2 (l). It is well known that the oxidation of CMH leads to the formation of the paramagnetic 3-methoxycarbonyl-proxyl nitroxide (CM•) [8].

Samples for calibration curves was obtained from a 10 mM stock solution of a standard: 3-carboxy-proxyl (CP•) (NOX-8.2, Noxygen, Germany) [9], [18] solved in KHB, and diluted to concentrations of typically 0, 5, 10, 25, 50, 100 and 500 µM. Exactly 100 µL of the calibration samples was transferred to 1 mL de-capped syringes and snap frozen in N2 (l).

**Figure 1:**
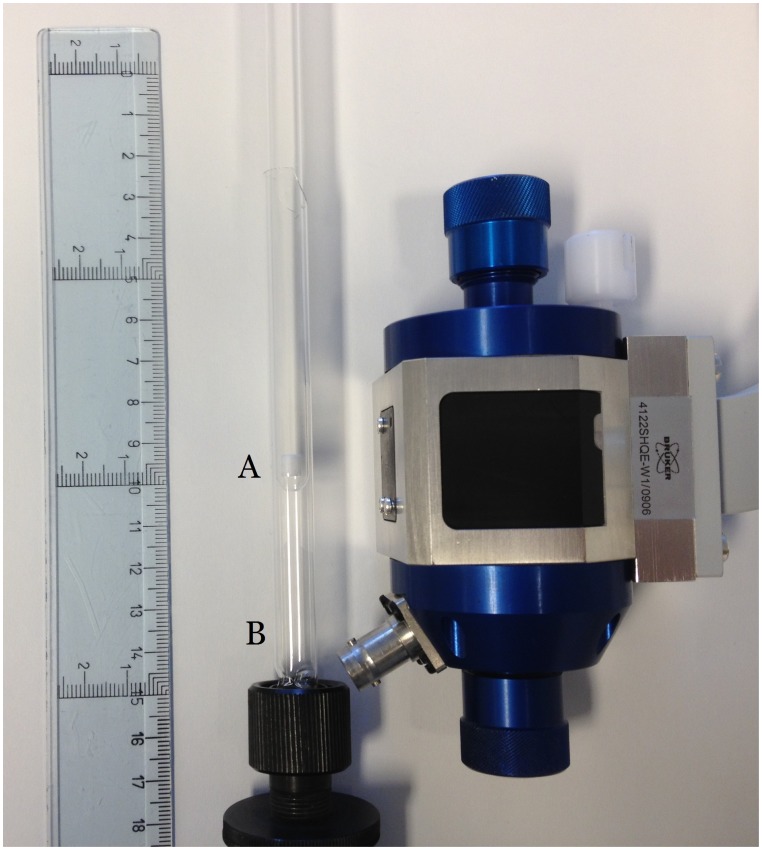
Photograph of the special made Dewar WG-821-TMR-SPECIAL (labelled “B”) containing a 100 µL tablet in a 8 mm precision bore Suprasil tube 513A-1PP-7SUP (labelled “A”). A Bruker 3122SHQE resonator is also shown in the figure indicating the position of the special made Dewar with the 100 µL tablet inside the resonator. Both the Suprasil tube and Dewar are from Wilmad-LabGlas (Vineland, New Jersey, USA).

EPR measurements were performed using a Bruker E5oo Elexsys X-Band EPR spectrometer equipped with a BRUKER ER 4122SHQE (in the following text referred to SHQE) resonator. All measurements were performed at 150 K using a Bruker ER 4111 VT variable temperature unit. The spectrometer settings were: 0.2 mW, 5 G modulation amplitude, 10 ms time constant, 20 s sweep time, 1024 measurement points, 600 G sweep width and 5 sweep added together for each measurement. At the time of measurement the sample (in its syringe) was removed from the storage Dewar and the syringe was warmed in the palms of the hands until a slightly thawed 100 µL “tablet” could be pressed out into a deep frozen 8 mm tube (precision bore Suprasil tube 513A-1PP-7SUP) which was immediately placed in a special made Dewar (WG-821-TMR-SPECIAL) in the EPR resonator maintained at 150 K. Both the Suprasil tube and Dewar are from Wilmad-LabGlas (Vineland, New Jersey, USA) and shown in Figure 1. No EPR signal could be detected for the empty sample tube or from PBS. The recorded EPR spectra were imported [10] into MATLAB (version R2011a, MathWorks, Inc.). Using an in-house developed MATLAB code all spectra were baseline corrected. The EPR signal intensity was determined as peak-to-peak height in the first derivative spectrum (*l*) (dimensionless).

Calibration samples of typically 0, 5, 10, 25, 50, 100 and 500 µM CP•, dissolved in KHB, were used to make a calibration curve for each measurement session. The relationship between EPR peak-to-peak signal intensity (*l*) and radical concentration *[ROS]* (µM) could be calculated using least-squares-method. The slope of the calibration curve was denoted as *s* (µM^−1^) and the y-axis intersection of the calibration curve was denoted as *l_0_* (dimensionless).

Radical concentration in each 100 µL frozen tablet could then be calculated using the calibration curve according to: *[ROS]* = (*l* – *l_0_*)/s.

The series of biopsies from rat myocardial tissue was analysed and then reanalysed under similar conditions after storage in −80°C during six months. For each of these two measurement sessions, a new series of calibration samples were made to ensure that a possible instability of the calibration samples should not influence the results.

## Results

**Figure 2:**
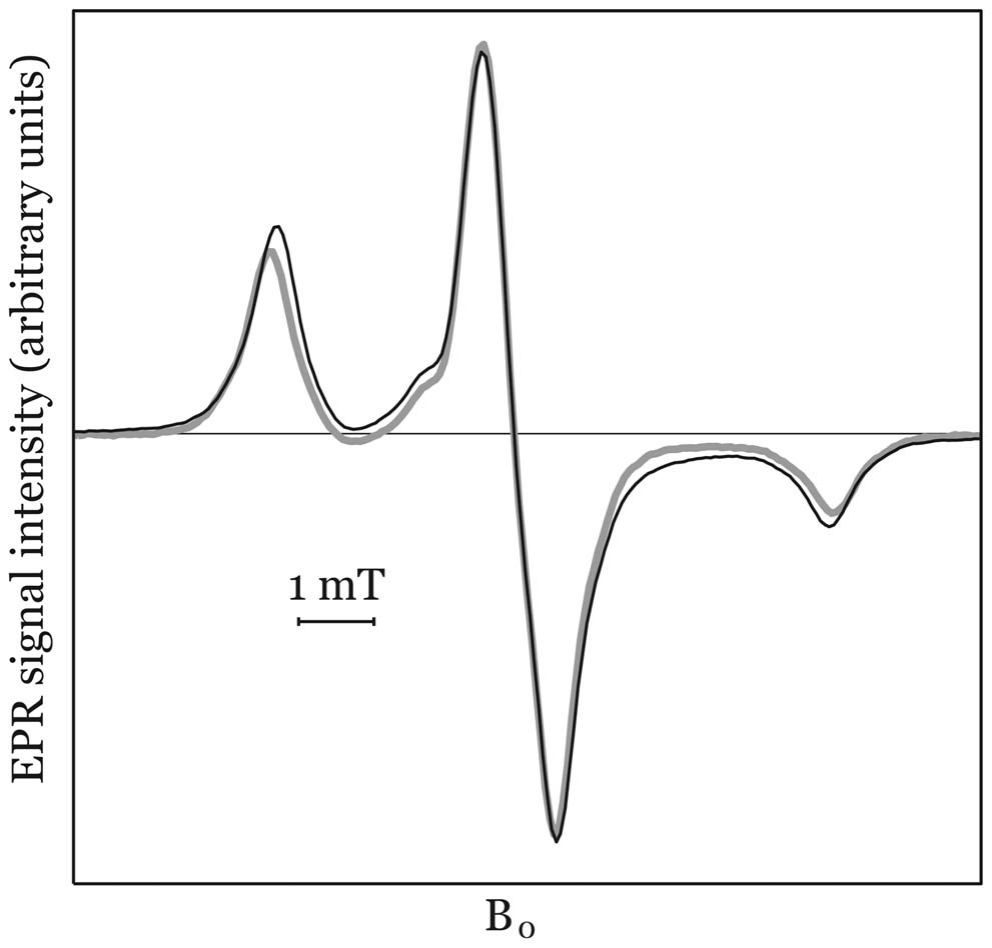
EPR spectra for CP• (grey line) and CM• (black line) obtained at 150 K using the parameters for quantitative EPR. For clarity only the central part of the spectra is shown and EPR spectra was normalised for equal signal intensity.

**Figure 3:**
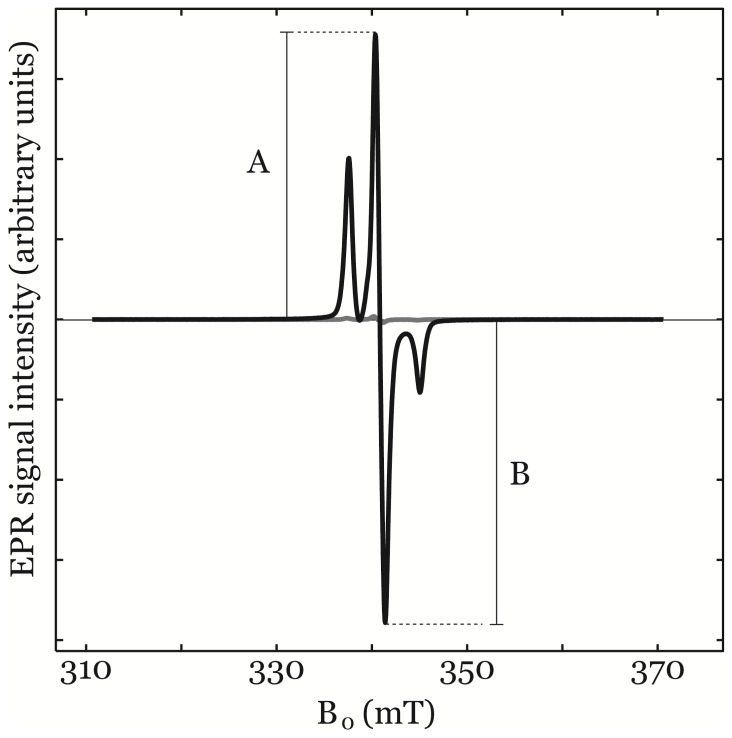
EPR spectra for calibration sample 500 µM CP• (black line) and 5 µM CP• (grey line) obtained using the parameters used for quantitative EPR. EPR signal intensity was defined as the peak-to-peak value A+B as indicated in the figure.

EPR spectra of CP• and CM• were identical at 150 K as shown in Figure 2. At 0.2 mW both samples were well within the linear relationship between peak-to-peak signal intensity and applied microwave power. The EPR spectra obtained at 150 K of 100 µL, 500 µM, CP• in KHB and 100 µL, 5 µM, in KHB are shown in Figure 3 (black and grey line respectively). The EPR signal intensity was defined as the peak-to-peak value A+B as indicated in Figure 3.

**Figure 4:**
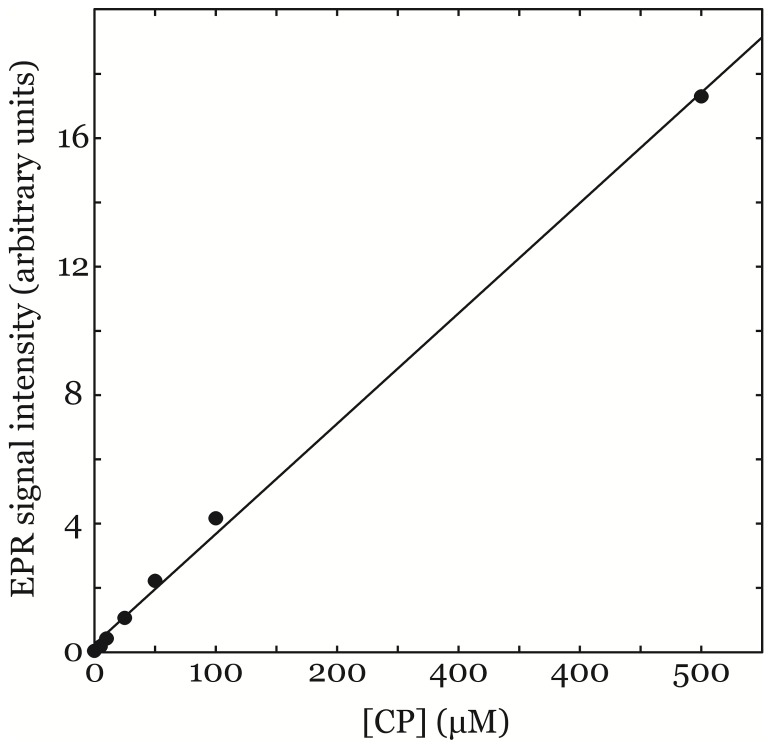
EPR peak-to-peak value as a function of [CP•] in calibration samples of CP• solved in KHB (circles). Calibration curve calculated using least-squares-method for the shown example was deduced to *s* = 0.03436 µM^−1^ (slope of calibration curve) and *l_0_* = 0.24179 (the y-axis intersection of the calibration curve (dimensionless)).

Calibration samples of typically 0, 5, 10, 25, 50, 100 and 500 µM CP•, dissolved in KHB, were used to make a calibration curve for each measurement session. A typical result is shown in Figure 4. For the shown example the slope of the calibration curve was deduced to *s = *0.03436 µM^−1^ and the y-axis intersection of the calibration curve was deduced to *l_0_* = 0.24179. Slope *s* and y-axis intersections *l_0_* of other calibration curves established for other measurement sessions are shown in Table 1. Measurement precision was estimated to ±10 µM (k = 1) based on the observed variance of the calibration samples in several different calibration curves obtained at different measurement sessions. Measurement accuracy of absolute radical concentration was estimated by repeated measurements on both tissue samples and additional samples of CP• dissolved in KHB at various concentrations and was estimated to be better than ±10 µM (k = 1).

**Table 1:** Slope *s* and y-axis intersections *l_0_* for calibration curves established for typical measurement sessions.

Measurement session#	*s*(µM^−1^)	*l_0_*(dimensionless)
1	0.03436	0.24179
2	0.06672	0.71693
3	0.08440	0.03488
4	0.02973	0.07082
5	0.07092	0.75417

In aortic endothelium from mice prone for atherosclerosis, the mean value of CM• was almost doubled compared to wild-type mice ([ROS]_mean_ = 112.5 µM as compared to [ROS]_mean_ = 67.5 µM), corroborating the use of this method in cardiovascular research.

A series of samples obtained from incubation of biopsies from rat myocardial tissue with CMH, was analysed and then re-analysed after storage in −80°C during six months. Results are shown in Table 2. No change in concentration of radical species could be observed after six months of storage. The calculated mean for both the initial experiment and the mean for the second experiment after six months of storage was [ROS] = 62 µM, where any possible difference between these measured values were found to be within the estimated uncertainty ±10 µM (k = 1).

**Table 2:** Table shows the initial results of quantification of ROS in rat myocardial tissue samples (first column) and the results of a repeated series of measurements after storage in −80°C during six month (second column).

Biopsy#	Initial measurements[ROS] (µM)	Measurements after storage at−80°C during six months [ROS] (µM)	Difference Δ(µM)
1	58	54	−4
2	57	56	−1
3	67	71	4
4	102	111	9
5	24	24	0
6	69	73	4
7	77	73	−4
8	56	51	−5
9	64	62	−2
10	59	60	1
11	83	82	−1
12	47	45	−2
13	33	36	3
14	34	34	0
15	97	98	1
16	64	60	−4

Third column show the difference between the two measurement sessions. (Estimated uncertainty ±10 µM (k = 1)).

## Discussion

Oxidative stress is defined as a physiological imbalance between the amount of ROS and antioxidant defence mechanisms and is linked to a large number of diseases including atherosclerosis [19], ischemia-reperfusion injuries [20], diabetes [21], cancers [22], and neurodegenerative diseases [23]. Recently, increased oxidative stress in patients undergoing cardiac surgery was demonstrated [24]. By using the present method, biopsies can easily be sampled during surgery and further processed in a distant lab giving new insight of how the biological response is altered in critical situations, such as during open-chest surgery. In this study, we used the spin probe CMH, an effective cell permeable membrane spin probe for the quantification of reactive oxygen species in vitro and in vivo. The concentration of the formed CM•, the spin probe adduct 3-methoxycarbonyl-proxyl nitroxide, is proportional to the concentration of the oxidant species that reacts with CMH since the spin probe is introduced after the tissue has been removed from the donor (CM•) [7], [8]. The free radical CP• with similar chemical structure as CM• constitutes a good reference sample as we expect similar solubility and relaxation behaviour as the accumulated CM• in the samples.

Standard methods for EPR spectroscopy of biological samples such as biopsies and cell suspensions includes measurements in room temperature or 37°C using special EPR flat cells, EPR tissue cells or capillaries. While measurements using any of these methods can be performed with high sensitivity and in addition be used to give important spectral information, it is often a challenge to use any of these methods to perform quantitative EPR spectroscopy with sufficient precision and accuracy to detect small, biological relevant, differences in generation of ROS. Even very small changes in sample position in the EPR resonator can introduce large differences in the resonator quality factor (Q factor) which subsequently introduces large differences in the detected signal intensity. These differences may be misinterpreted as a difference in the amount of ROS. The use of a reference sample such as Mn^2+^/MgO or Ruby can reduce the errors associated with changes in spectrometer sensitivity, but signal normalisation to a reference sample is associated with an additional uncertainty that limits the precision that can be achieved. Recent developments of reference-free quantitative EPR spectroscopy opens for the possibility of quantifications with an error less than +/−10% (k = 1) without the use of any reference [12], [13].

However, for many biological applications it is desired to observe even smaller differences in e.g. generation of ROS. In this work we present a new method for quantitative EPR with higher precision and accuracy than what is available with standard methods for quantitative EPR. The proposed method is based on a recently developed method for EPR dosimetry [16], [17] where high precision and accuracy could be achieved without the use of an external reference by precise control of sample size, sample position and the use of a sample tube with a homogenous wall thickness. By making frozen “tablets” with identical size and volume and by performing the EPR measurements at 150 K we were able to use the methods developed for EPR dosimetry to performed quantitative EPR of ROS in biopsies with high accuracy and precision.

Preliminary experiments showed that measurements at 150 K was a good compromise between the high signal-to-noise ratio achievable at low temperatures and a reasonably low nitrogen flow in the variable temperature unit to avoid loss of accuracy and precision due to sample tube movements due to extensive flow of nitrogen. An obvious draw-back of performing EPR measurements at low temperatures (e.g. 150 K) is that the additional spectral information that may be extracted at room temperature or above (e.g. rotational dynamics and hyperfine structure) is lost in the slow motion EPR spectra that can be observed at low temperatures. The method proposed in the present manuscript is therefore not suitable to use in combination with spin traps such as DMPO [25], and DEPMPO [26] when it is of interest to distinguish generation of e.g. superoxide radicals from hydroxyl radicals using the slight differences in the EPR spectra that can be observed for the different spin trap adducts. However, spin probes that can detect several forms of radicals, such as the CMH, must be used in combination with scavengers or inhibitors, since increased activity of the enzyme NADPH oxidase will raise the production of the superoxide ion, while overexpression of e.g superoxide dismutases, will hamper the EPR signal [5], [27]. Since the superoxide ion represents the majority of radicals in vivo and in vitro, the action of endogenous superoxide dismutases will inhibit the CMH signal by 90% [27], [28]. The highest interaction constant with CMH is the superoxide ion, but CMH also reacts with peroxynitrite, nitrogen dioxide, and the peroxyl radical [5], [8]. Although the interaction with hydrogen peroxide seems to be negligible [7].

Spin probes are mainly used to quantify the extra and intra cellular amount of radicals that can oxidase CMH and no changes in the EPR spectra is observed for reactions with different types of ROS in contrary to spin traps [3]. Measurements at low temperatures do therefore not come at the cost of loss of interesting spectral information when using spin probes, since the EPR spectra of the spin probe adduct is well-known at room temperature and contains no biological relevant information.

The standard technique for EPR signal intensity quantification is by double integration of the 1^st^ derivative EPR line shape. As is well known, it is important to extend the EPR sweep width far beyond the line-width of the signal that is to be integrated to avoid errors. We therefore choose to perform the measurements presented here using 600 G sweep width. However, our experiments showed that even higher precision could be achieved when using the simple peak-to-peak signal intensity in the 1st derivative EPR spectra as compared to double integration of the 1st derivative EPR line shape. The results presented here are therefore based on measurements of peak-to-peak signal intensity in the 1st derivative EPR spectra. Higher signal-to-noise ratio and possible also higher measurement precision in quantification of ROS can probably be achieved using a more narrow sweep width which is more suitable for measurements of signal intensity as peak-to-peak signal amplitude when there is no need to extend the EPR sweep width far beyond the line-width of the signal. This will be studied in a future work.

Due to the extremely short lifespan of ROS, it is of utmost importance to measure ROS at the place of its origin. Increase of ROS contributes to oxidative damage, but ROS in small concentrations are important as signalling molecules in normal considerations, and may also contribute to pathological development. To establish the precise role of ROS requires the ability to accurately quantify the amount of ROS as well as the oxidative damage [1]. To test the present method, the concentration of ROS was quantified in the aortic arch from eight mice with (N = 6) or without (N = 2) aortic plaque. In the collected biopsies, which were as small as 6 µg, we could use the current method to accurately quantify the amount of ROS even if the tissue had been frozen and stored. Our results showed that the content of reactive oxygen species that can oxidase CMH was increased in the aortic endothelium from atherosclerotic mice. We therefore conclude that the proposed method may be useful in especially cardiovascular research where the mouse aorta is central for atherosclerotic lesions.

In comparison to quantifications of ROS in e.g. human lymphoblast cell lines [6] or mice vascular smooth muscle [27] this study demonstrates higher absolute concentrations of ROS. However, another study [29] describing quantification of CM• in human blood reports concentrations in the same order of magnitude as in the present study. The range of expected concentration of CM• in mice atherosclerotic plaques incubated during 60 minutes have not, to the best of our knowledge, been fully established and may be a subject for future studies. Since oxygen radicals in general are short lived molecules an increased incubation time might be necessary to evaluate the capacity of the involved enzymes during pathogenesis. Here, the authors aimed to evaluate the stability oxygen radicals in frozen and stored biological samples, and therefore refinement of the used spin probe was not the main focus.

Measuring reactive oxygen species is highly useful in several biological and medical research areas such as in cancer and cardiovascular dysfunction. Also, before recommending dietary antioxidants measuring oxidative stress can be useful [30]. However, production of ROS is highly individual and depends on the external and internal stress of the organism [31]–[33]. This means that it is hard to control the amount of internal ROS and antioxidant production in the organism, or the stress added by anaesthesia and the surgery. However, by shorten the time in anaesthesia, and by limiting the time to tissue preservation, ROS measurements will be more feasible and accurate. The present method introduces a possibility to incubate and measure frozen tissue under similar conditions, and thereby reducing day-to-day artefacts.

The access to EPR spectroscopy and necessary laboratory equipment is in general a limitation, and traditional spin probes have been showed to have limited stability during storage and transport [34]. We show here, for the first time, that biological samples can be collected and stored for future incubation with spin probe, and also further stored before EPR analysis, without loss of signal intensity. This method can deepen our knowledge of the complexity between ROS and antioxidants. Moreover, the same biopsies can be measured repeatedly, and comparisons of measurements between different research groups are now possible, further improving the quality of measurements.
